# 3D segmentation of plant root systems using spatial pyramid pooling and locally adaptive field-of-view inference

**DOI:** 10.3389/fpls.2023.1120189

**Published:** 2023-04-04

**Authors:** Jonas Alle, Roland Gruber, Norbert Wörlein, Norman Uhlmann, Joelle Claußen, Thomas Wittenberg, Stefan Gerth

**Affiliations:** ^1^ Fraunhofer Institut für Integrierte Schaltungen (IIS), Fraunhofer Institute for Integrated Circuits Institut für Integrierte Schaltungen (IIS), Division Development Center X-Ray Technology, Fürth, Germany; ^2^ Friedrich-Alexander-Universität Erlangen-Nürnberg, Chair for Visual Computing, Erlangen, Germany; ^3^ Fraunhofer Institut für Integrierte Schaltungen (IIS), Fraunhofer Institute for Integrated Circuits Institut für Integrierte Schaltungen (IIS), Division Smart Sensors and Electronics, Erlangen, Germany

**Keywords:** root phenotyping, root system analysis, computed tomography, weakly supervised learning, sub-labels, scale invariance, flood-filling, convolutional neural networks

## Abstract

**Background:**

The non-invasive 3D-imaging and successive 3D-segmentation of plant root systems has gained interest within fundamental plant research and selectively breeding resilient crops. Currently the state of the art consists of computed tomography (CT) scans and reconstruction followed by an adequate 3D-segmentation process.

**Challenge:**

Generating an exact 3D-segmentation of the roots becomes challenging due to inhomogeneous soil composition, as well as high scale variance in the root structures themselves.

**Approach:**

(1) We address the challenge by combining deep convolutional neural networks (DCNNs) with a weakly supervised learning paradigm. Furthermore, (2) we apply a spatial pyramid pooling (SPP) layer to cope with the scale variance of roots. (3) We generate a fine-tuned training data set with a specialized sub-labeling technique. (4) Finally, to yield fast and high-quality segmentations, we propose a specialized iterative inference algorithm, which locally adapts the field of view (FoV) for the network.

**Experiments:**

We compare our segmentation results against an analytical reference algorithm for root segmentation (*RootForce*) on a set of roots from Cassava plants and show qualitatively that an increased amount of root voxels and root branches can be segmented.

**Results:**

Our findings show that with the proposed DCNN approach combined with the dynamic inference, much more, and especially fine, root structures can be detected than with a classical analytical reference method.

**Conclusion:**

We show that the application of the proposed DCNN approach leads to better and more robust root segmentation, especially for very small and thin roots.

## 1 Introduction

To optimize and selectively breed resilient and sustainable plant species, automatic or interactive segmentation subterranean root structures from reconstructed CT scans is of great importance for phenotyping plants (Mooney et al., 2012; Atkinson et al., 2019). Thus, the quantification of the root-system architecture reaction on biotic and abiotic stress factors is possible. Up to now, most optimization and breeding efforts have relied on phenotyping of above ground structures and completely ignored ‘below-the-ground’ structures.

**Figure 1:**
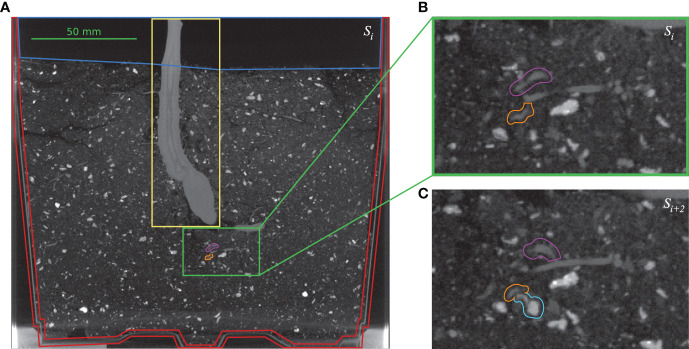
Challenges for small root segmentation: **(A)** one vertical 2D slice *S_i_* from volume *V*_5_ (see **Table 1**). Yellow box: stem of the cassava plant; red region: planting pot; dark blue region: air; green box: example of soil mixture; magenta and orange marks: two branches of a very thin root. **(B)** Enlarged version of the green box in **(A)** with marked root branches. **(C)** Adjacent slice *S_i+_*_2_ where the lower root branch (orange) passes a small stone (cyan) with a similar appearance.

In contrast to many conventional methods, such as soil-coring, root washing or *shovelomics* (Trachsel et al., 2011), the non-invasive scanning and reconstruction of CT-volumes of roots and their successive 3D segmentation is a crucial and powerful approach in this field of research [e.g. De Smet et al., 2012; Mooney et al., 2012; Metzner et al., 2015; Ahmed et al., 2016; Atkinson et al., 2019). However, these new possibilities for the non-destructive monitoring of root systems demand high quality data sets of segmented data for quantitative data analysis. This is important since a classical ground truth *via* root excavation and washing, results in a loss of fine root structure and interconnectivity of the whole root system. To this end, the 3D segmentation of the root structures was mainly performed by analytical algorithms based on classical image processing and image analysis methods [e.g., Mairhofer et al., 2011; Flavel et al., 2012; Mairhofer et al., 2015; Flavel et al., 2017; Gao et al., 2019; Soltaninejad et al., 2020; Gerth et al., 2021; Phalempin et al., 2021; Ferreira et al., 2022; Lucas & Vetterlein, 2022), which, however, are not able to detect roots on all scales equally. See **Figure 1** for an example which contains challenging small and fine roots.

To overcome the limitations of the current analytical methods, we propose an improved deep neural network based approach, which is (a) able to adapt to different feature scales of the roots to be delineated, (b) can be trained with noisy information, and (c) furthermore is able to efficiently handle large scale 3D data sets of roots during inference. We make use of a classical deep convolutional neural network (DCNN) architecture which is enhanced by a ‘Spatial Pyramid Pooling’ (SPP) layer (He et al., 2014) (see Section 4) to gain scale invariance for root segmentation by supporting arbitrarily sized ‘*field of views*’ (FoVs) (see Section 3.4) as input sample and is furthermore using a weakly supervised training scheme (Khoreva et al., 2017) (see Section 5).

The work is structured as follows: Section 2 gives an overview of related work, while Section 3 introduces the 3D volumetric CT data of plant root-structures used for training and testing of the modified network. Section 3 also includes information about the labeling of the needed ground truth from an analytical approach, motivates the usage of size varied FoVs, and explains the proposed sub-labeling technique yielding a fine-tuned reference data set.

Section 4 introduces the proposed architecture of the deep neural network including the spatial pyramid pooling layer. In Section 5 the novel training approach is presented including a description of the weakly supervised training loop.

The novel inference algorithm is explained with a detailed walk-through of its functionality in Section 6. Experiments and results are presented in Section 7. Finally, a critical discussion is given in Section 8 and the work is concluded in Section 9.

## 2 Related work

The challenging task at hand consist of a semantic segmentation of each voxel within a data volume (acquired from reconstructed CT scans) into the two labels *L* ∈ {*‘root’, ‘non-root’*} denoting either *‘root’* being foreground or *‘non-root’* being background voxels denoting surrounding soil, air, or the planting pot (cf. **Table 1**). The underlying challenge of this binarization relates to the strong variance of the root diameters, which range from the storage root diameter of Ø = 10 – 30 mm, to lateral root extensions with diameters in the range of Ø = 0.2 mm, as well as varying scanning conditions.

**Table 1:** Typical examples of different *cassava* root systems, 3D-segmented with *RootForce* (Gerth et al., 2021).

Training				Validation
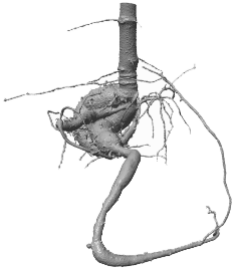	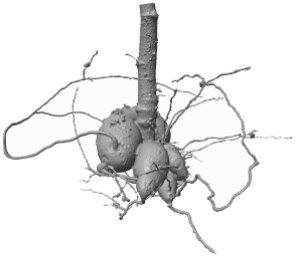	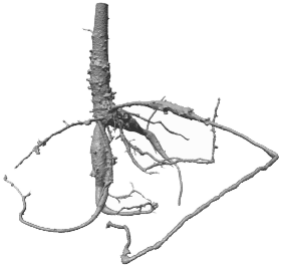	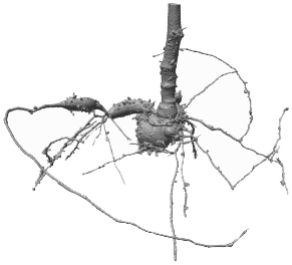	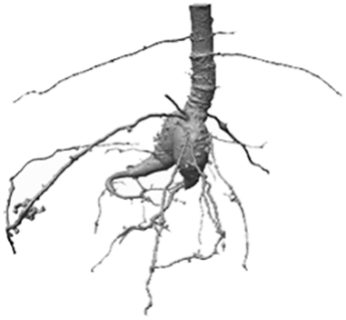
*V*_1_: 4,264,632 voxels	*V*_2_: 10,462,217 voxels	*V*_3_: 1,596,662 voxels	*V*_4_: 3,577,330 voxels	*V*_5_: 2,834,479 voxels

In the past years various methods and algorithms addressing this task have been proposed. See (Xu et al., 2018; Li et al., 2022) for an excellent broad and deep overview of the research area. These can be clustered in two groups, namely (a) traditional knowledge-driven top-down image-processing and analysis methods, and (b) more recently developed data-driven bottom-up approaches employing deep neural network architectures and a large corpus of labeled training data.

Examples of the first category include the ‘*RootForce*’ algorithm (Gerth et al., 2021), which solves this task by analyzing the curvature of the gray value profile of the root voxels using classical image processing and analysis approaches. Using the specific mean gray values of the two object types ‘*root*’ and ‘*non-root*’, *RootForce* sorts out all objects which are not in a specific gray value range and thereby generates a mask to indicate possible root voxels. Based on this mask, Frangi’s vesselness approach (Frangi et al., 1998) is applied to create two root volumes for small and large roots, respectively.

Another analytical approach, referred to as *‘Rootine’*, also building upon Frangi’s vesselness filter was presented by Gao et al. (2019) and Phalempin et al. (2021), and uses 3D hysteresis thresholding to binarize the resulting image. A similar approach employing region and volume growing method has been proposed by Flavel et al. (2012) and later published under the name ‘*Root1*’ (Flavel et al., 2017).

Further analytical approaches have been suggested by Mairhofer et al. (2011), who have developed the so-called *‘RooTrak’* algorithm. This approach makes use of slice wise level-set techniques combined with the so-called *Jensen-Shannon* divergence to segment plant roots in volumetric data sets. Later, this procedure was expanded to enable the tracking of multiple touching roots using the iterative closest point (ICP) algorithm to match and separate multiple possible roots of different root systems between slices (Mairhofer et al., 2015).

The second group of methods relate to deep-learning inspired approaches, which have recently been introduced to this field of research, e.g., a multi-resolution encoder-decoder network to handle the diverse scales of root systems was proposed by Soltaninejad et al. (2020). They propose a parallel processing pipeline featuring a path of high resolution with a small receptive field and a path of lower resolution but large receptive field to segment 3D volume patches. This approach has been built on top of the well-known U-Net architecture (Ronneberger et al., 2015; Cicek et al, 2016) used for semantic segmentation.

Smith et al. (2020) also employ a U-Net to automatically segment *rhizotrons* in RGB photographs of *Chicory* root systems. Here, the network was trained and validated on a set of 50 manually labeled 2D images and compared to a baseline using Frangi’s vesselness filter (Frangi et al., 1998). Using 3D-MRI data, the work by Zhao et al. (2020) also utilized a U-Net architecture for the segmentation of roots using custom loss modifications to reduce erroneous disconnected roots.

Alternatively, Shen et al. (2020) make use of a slightly modified *DeepLabv3+* network architecture (Ayhan, 2020) to segment RGB photographs of cotton root systems. Specifically, they introduced a sub-pixel convolution to the up-sampling path of the model and compared their results with manually annotated data as well with the results of a U-Net based segmentation. Thesma and Mohammadpour Velni (2023) propose a conditional generative adversarial network (cGAN) to augment their available root data set of RGB photographs from plant roots grown in clear gel. They further used a SegNet trained on both synthetic and real data for segmentation of fine root systems.

Our own proposed network (see **Section 4**) builds upon the work by He et al. (2014), who introduced the so-called ‘Spatial Pyramid Pooling network’ (‘SPP-network’) with the goal to alleviate the fixed size constraint of input images for classical deep convolutional neural Networks (DCNNs) (Schmidhuber, 2015; Krizhevsky et al., 2017). After feature extraction with several convolution layers and by introducing a pooling method, that generates fixed size outputs from arbitrary sized inputs a pyramid of feature maps is generated, ranging from a coarse-to-fine detail level. This allows training a network with input data of varying image sizes, resulting in a more robust classification as output with respect to scale variance. Specifically, for this contribution we employed this SPP-net (see **Section 4**) and extended it to the domain of CT-data and 3D root segmentation.

## 3 Data

The data set used for this project was generated from fourteen high-resolution CT root scans from different potted *Cassava* plants with the same set of scanning and calibration parameters. From the projective raw data, cuboid volumes with spatial dimensions of 17.9 cm × 17.9 cm × 15.7 cm were reconstructed, and with a voxel edge length of 175 μm resulted in data volumes of 1024 × 1024 × 900 voxels per plant. For more details about the scanning see Gerth et al. (2021)

### 3.1 Data description

**Table 1** provides typical examples from the fourteen scanned and 3D-segmented root systems of cassava plants. The bottom row lists the count of detected root voxels within each volume. The 3D-segmentations of the depicted root structures have been generated by the *RootForce* algorithm (Gerth et al., 2021) with a vesselness filter in the range of 0.2–0.5 mm resulting in a volume of small roots, and furthermore used a 3D-Gauss filter (σ = 0.5mm) to support the extraction of large roots (see **Section 2**). Both obtained volumes are binarized and merged yielding the final binary root segmentation.

**Figure 1A** shows one vertical 2D image slice *S_i_* extracted from volume *V*_5_. This side view depicts the large (bright) stem of the cassava plant (denoted by the yellow box in the top center) surrounded by the (dark) soil mixture, the planting pot below (red region) and the air above (dark blue region). The soil mixture is rather inhomogeneous with many components of differing gray values, including small pebbles. To enhance visibility of the very thin root structures depicted in the soil, the contrast was increased. One challenge in this exemplary data is highlighted in the green boxes in **Figure 1A** and enlarged **Figure 1B**, where two branches of a very thin root (orange and magenta) are denoted. **Figure 1C** depicts the two branches of the same root in the adjacent image slice *S_i+_*_2_, (shifted two voxels perpendicular to the slicing plane) passing a small stone (cyan) with very a similar roundish structure and gray level values.

### 3.2 Data split

For the training, validating, and testing of the proposed SPP-network (see **Section 4**) the fourteen CT scans were divided. Four volumes (*V*_1_ – *V*_4_) were used as training data and one volume (*V*_5_) for the validation of the deep neural network. The remaining nine (*V*_6_ – *V*_14_) scans were used for testing. The training volumes were chosen carefully with the distinct aim of providing a diverse set of root examples despite the otherwise identical physical parameters.

### 3.3 Labeled training data

When training a DCNN to delineate the complex root structures into the fore- and background labels *‘root’* and *‘non-root’*, a sufficient amount of adequately labeled ground truth data is required. However, there is only limited availability of training data with a satisfactory image and label quality, thus hindering the training process. Since manual annotation of such CT data is time consuming and error prone, segmentation results of the training data *D* = *{V*_1_, *V*_2_, *V*_3_, *V*_4_ *}* (see **Section 3.2**) obtained from the *RootForce* algorithm were used as label volumes *L*_0_ (see **Table 1**) in an *initial* training data set *T*_0_ = (*D*, *L*_0_). The deep neural network (see **Section 4**) was trained by a weakly supervised learning approach, as proposed by Khoreva et al. (2017), to recursively refine the data set. During the weakly supervised learning iterations, the segmentation results of the network *N*_0_ from the first training iteration are used as label volumes for subsequent training iterations, see **Section 5** for details.

### 3.4 Sample fields of view

Due to memory restrictions, an entire volumetric 3D scan of the roots (with a total of 1024 × 1024 × 900 voxels) cannot be processed at once. Therefore, we consider small sub-volumes or small 3D-patches from the complete volumes as input samples. Even though only the center voxel of each patch is classified into either label ‘*root*’ or ‘*non-root*’, the surrounding volume patch provides important contextual information. Consequently, the reference label of one sample is *not* a classification of the whole volume patch, but a binary label for the center voxel, encoding either ‘*root*’ or ‘*non-root*’.

In order to detect thin *and* thick roots (see **Table 1**; **Figure 1**) with equal accuracy, similar structural root features need to be represented across scales. To restrict the introduction of additional noise and expensive preprocessing during inference, scaling techniques utilizing filtering were avoided. Instead, as all samples originate from image data with the same voxel resolution, we vary the edge length of the patches for each sample; hence, the training sample size of an input patch varies only in the voxel count and thus the dimensions of the cuboid, but not in the physical voxel size. In the remains of this work, we will refer to the varying sample size as ‘*field of view’* (FoV) which is created by extracting the neighboring region around each voxel. This region can extend between *r* = 2 to *r* = 7 voxels in each direction, hence resulting in cubic samples with edge lengths in the range from *l* = 5 to *l* = 15 voxels.

### 3.5 Sub-labeling

Since every volumetric data sample corresponds to a distinct voxel, which is either of class ‘*root’* or class ‘*non-root*’, a naively constructed training data set would inherit a skewed class distribution, as there are much less root voxels than non-root voxels present in each CT scan. Randomly drawing samples from the pool of possible non-root voxels to match the count of root voxels, could yield many trivial negative samples, such as volume patches depicting only soil or air around the plant pot, see **Figure 1A**. To provide an adequate training data set with an ideally uniform class distribution, the applied data preprocessing includes a categorization of the training and validation samples into sub-labels, which distinguish the center voxel of the volume patch from the surrounding voxels.

Because during the later inference, the proposed network (see Section 4) shall be provided a volume patch, and its output should predict one of the classes ‘*root*’ or ‘*non-root*’ for *only* its center voxel, it is important to train the network with a sufficient set of ‘*difficult negative*’ examples. Specifically, in such ‘difficult negative’ samples, some root voxels should be present in the volume patch, while the center voxel *is not* of the class ‘*root*’. In this way, the network will be prevented from classifying the volume patch as a whole and instead is taught to differentiate between the contents in the center voxel and its surrounding.

Furthermore, another challenge with respect to the application of deep convolutional neural networks to plant root segmentation is the misclassification of voxels depicting the plant pot as class ‘*root*’. As the non-trivial geometry of the pot base (see **Figure 1A**) could not be easily extracted or excluded by a mask, the sub-labels also differentiate within the *‘non-root’* class, namely between *‘sediment’* and *‘else’* contents, where ‘*else*’ is used as collective term for all voxels depicting the plant pot or air. This approach allows further specification for the included samples of the negative ‘*non-root*’ class in the training and validation data. For example, ‘*non-root*’ samples, which contain roots in the surrounding but not in the center voxel and are located next to the edges of the plant pot.

**Figure 2:**
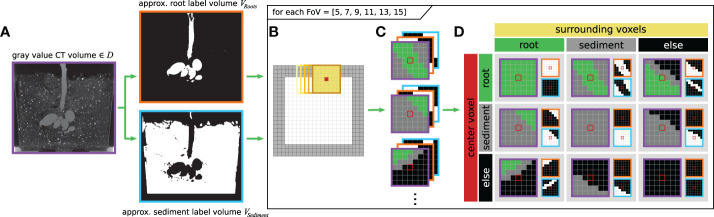
2D description of the volume sample categorization into nine sub-labels (*‘root-root’*, *‘root-sediment’*, *‘root-else’*, *‘sediment-root’*, *‘sediment-sediment’*, *‘sediment-else’*, *‘else-root’*, *‘else-sediment’* and *‘else-else’*). **(A)** input data and pre-labeled masks *V*_Roots_ and *V*_Sediment_ corresponding to one scan; **(B)** extraction of individual volume patches according to the current FoV for each interior voxel; **(C)** example of the depicted contents within the volume patches; **(D)** overview of possible sub-label combinations differentiating between combinations of content in the center voxel and the surrounding voxels.

As depicted in **Figure 2**, for the categorization of the training samples into adequate sub-labels, two label volumes *V*_Roots_ and *V*_Sediment_ are employed (**Figure 2A**): The first volume, *V*_Roots_, is a binary volume and depicts an approximate labeling *V*_Roots_ = *L_i_* of the ‘*root*’/’*non-root*’ classification, which in the first approximation is obtained from a *RootForce* segmentation (see Section 3.3). The second label volume *V*_Sediment_ approximates a sediment segmentation, which is generated from the inverse *V*_Roots_ label volume. Additional image processing steps are applied in order to remove air pockets in the soil and the plant pot from the sediment labeling. However, a conservative box-mask, which also excludes several sediment voxels, was needed for the lower volume due to the complex geometry of the pot bottom. Hence, it is assured that no voxels from ‘pot’ or ‘air’ are contained in V_Sediment_, and thereby the sediment label volume helps differentiating between the content classes ‘sediment’ and ‘else’. The original gray level CT-volume, depicted in **Figure 2A**, only serves for a better description of the process, as its information is not needed to generate the sub-labels.

The following steps are repeated for all possible radius sizes *r* ∈ {2, 3, 4, 5, 6, 7} of the FoV: For every ‘interior voxel’ of both the approximated ground truth volume *V*_Roots_ as well as the approximated sediment label volume *V*_Sediment_, sample patches, which are centered on the current voxel and sized according to the current radius *r*, are extracted and then used to categorize each sample. The denotation of ‘interior voxels’ refers to those voxels, which are at least *d* = ⌊*r*/2⌋ voxels away from the volume’s edges, such that the FoV can be placed around the voxel without needing to pad the volume.

**Figure 2B** depicts the interior voxels of the volume in *white*, while for the FoV the center voxel is marked in *red*, and *yellow* indicates the voxels in the surrounding regions. Exemplary resulting patches are shown in **Figure 2C**.

As motivated earlier, the categorization of all volume patches into sub-labels shall differentiate the occurring content combinations of the center voxel and the surrounding voxels, where the content classes are *‘root’*, *‘sediment’*, and *‘else’* – surrogating ‘pot’ and ‘air’. **Figure 2D** shows the considered sub-labels in a table-like structure, where the contents of the center voxel are organized along the rows and the contents of the surrounding voxels are organized along the columns. This is also reflected by the respective color-coding of **Figure 2B**. The secondary table headers are color-coded to match the three content types (*‘root’*, *‘sediment’* and *‘else’*). In total nine different combinations of sub-labels are considered. The first row contains the sub-labels, which correspond to the original ‘*root*’ class, while all other sub-labels are of the ‘*non root*’ class. Each table entry gives an exemplary depiction of the three volume patches (edge colors indicate the affiliations to the source volumes). With the two binary volume patches, *V*_Roots_ and *V*_Sediment_, and some binary logical operators, every sample can now be categorized into a unique sub-label. The output of this process is a table for each FoV radius size *r* ∈ {2, 3, 4, 5, 6, 7}, containing the total amount of available samples per sub-label and storing the respective sample coordinates.

By specifying the sample counts per sub-label, a class-balanced training data set can now be constructed. To this end, we define a distribution, dependent on the difficulty and availability of each sub-label.

This above-described procedure of sub-label generation is repeated for all five CT scans used as training or validation volume. Where possible, similar distributions for every volume and FoV combination were used. However, this goal is not always attainable since the availability of the sub-label depends on the spatial extend of the addressed root system and the FoV radius *r*. Specifically for the later, the proportion of samples containing sub-labels, which encode for a mix of content types, scales proportionally with the FoV size.

## 4 Deep neural network architecture

**Figure 3:**
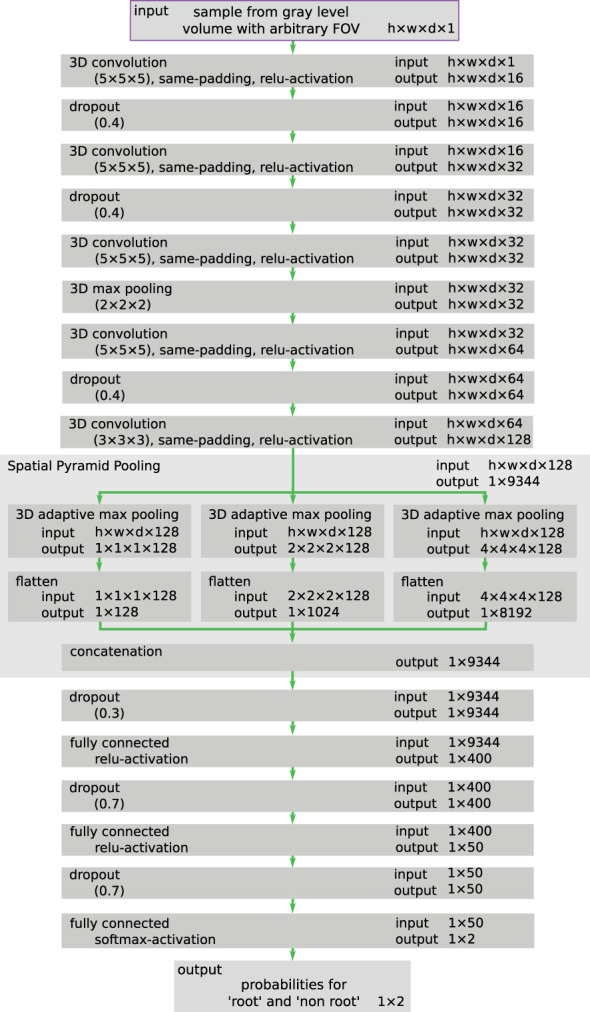
Schematic depiction of the employed network architecture. Specifically, the spatial pyramid pooling layer in the center of the network consists itself of multiple internal layers, which are processed in parallel. As the input can be of arbitrary sizes, the input and output dimensions are generically denoted as *h* × *w* × *d*.

For our experiments we employ a classical deep convolutional neural network (DCNN) structure (Schmidhuber, 2015; Krizhevsky et al., 2017) with a fully connected layer at the end as depicted in **Figure 3**.

However, one known downside of this network structure is the lack of sufficient scale invariance. This is a crucial issue as due to their fractal geometry (Dannowski & Block, 2005) the root structures depict strong similarities across different scales. Hence, the DCNN needs to learn scale invariant features, which can be reused on different scales to detect all types of roots robustly. This necessity is incorporated into the DCNN by including a ‘Spatial Pyramid Pooling’ (SPP) layer (He et al., 2014). This SPP-layer enables the network to learn and generalize scale-invariant features, and furthermore, relieves the size constraint of the input samples, which is inflicted by the fully connected layers. In order to utilize both advantages, different sized volume patches – also referred to as ‘*fields-of-view*’ (FoVs) (see **Section 3.4**) – which are created in the sub-labelling process (see **Section 3.5**) - are used as input for training of the same network.

The DCNN incorporates a total of five successive convolutional layers with an increasing count of channels to extract characteristic features from the gray level input sample *via* 5 × 5 × 5 kernels.

The subsequent spatial pyramid pooling (SPP) layer (He et al., 2014) consists of three parallel adaptive pooling layers, each generating a feature description on a different fixed scale. The sliding kernel of each pooling layer changes its size adaptively to ensure fixed output dimensions. Thereby, the total complexity of the feature description remains constant for arbitrarily sized inputs. For this application a pyramid consisting of the three levels is used, with each level having a cubic output shape with edge lengths of 1, 2, and 4 voxels, respectively.

After flattening and concatenation, the output of the SPP-layer is forwarded into the first layer of a classical Multi-Layer Perceptron (MLP). Three fully connected layers reduce the extracted feature vector to a final prediction of the two classes *‘root’* and ‘*non-root*’. The last layer of the DCNN uses the *softmax* activation function to scale the output value to the probability of belonging to the respective class.

For regularization purposes, dropout (Srivastava et al., 2014) is used with a probability of 40% after the convolution layers and 70% after the fully connected layers. ReLU is employed as activation function.

## 5 Network training

Within the scope of this work, a so-called *weakly supervised training* scheme as proposed by Khoreva et al. [^26^], is employed by using the available CT training data (Section 3.1) with *iteratively* improving label data from *RootForce* (Section 3.3). In each iteration, the sub-labeling (Section 3.5) is used to balance the data set, and a dynamic inference algorithm (Section 6) generates the improved labeling.

Within the *weakly supervised learning* approach, a network model *N_i_* is trained on an iteratively improving training data set *T_i_ = (D, L_i_)*. It has been observed by Khoreva et al. **[** ^26^ **]**, that when re-applying the network model *N*_i_ on the training data D, the output *N_i_* (*D*) *= O*_i_ of the network captures the shapes of the objects (in this case the root structure) significantly better than the label data during the training. This has inspired a recursive training procedure, where the achieved output labels replace the training labels *L_i+1_* = *O_i_* and together with the original training data *D* serve as a new training data set *T_i_*_+1_ = (*D*, *L_i_*_+1_) = (*D*, *N_i_* (*D*)) for another round of training.

Due to the generalizing capabilities of deep neural networks, incorrect outlier-labels become more aligned to the majority of correct labels over the course of recursively re-evaluating the training data with the neural network. Hence, a robustly trained neural network will smooth out possible label noise and thus yield an improved labeled training data set *T_i_*_+1_ = (*D*, *L_i+_*_1_) = (*D*, *N_i_* (*D*)). However, the weakly supervised learning paradigm cannot guarantee an improvement. Mistakes in the label data will not be removed generally, but depending on the training scheme and data set, label noise can be reduced by smoothing of the data-label correspondences. An amplification of mistakes in the training data set, resulting in ‘*label drift*’, was not observed for this work. While difficult to verify, the fine-tuning of the training data set *via* sub-labeling (see Section 3.5) might steer the training process towards greater robustness and thereby avoid label drift. The recursive training-evaluation cycle was repeated three times, each starting with a new network model *N_i_* from scratch.

**Figure 4:**
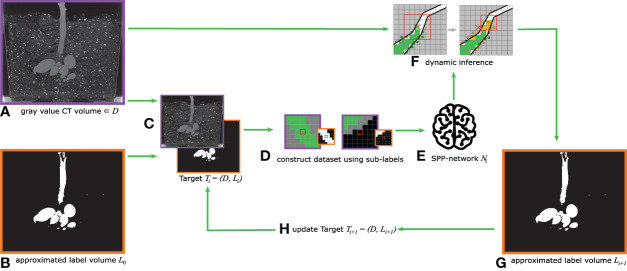
Schematic overview of the weakly supervised training loop. From gray value CT data *D* **(A)**, together with corresponding label data *L_i_* (provided by the *RootForce* algorithm for *i=0*) **(B)**, the training data *T_i_* = (*D*, *L_i_*) **(C)** is used to construct a sub-label balanced data set **(D)** which is then used to train a SPP-network model *N_i_* **(E)**. The trained SPP-network *N_i_* is applied on the training data *D* using the proposed dynamic interface **(F)**, yielding a new labeling *L_i+1_* **(G)**. In the next training iteration, this output is used as new (and improved) reference label *L_i+1_* **(H)**.

**Figure 4** summarizes the training iterations of the weakly supervised learning scheme of the proposed SPP-network (**Figure 4E**). As mentioned, the first iteration *i = 0* uses the segmentation calculated by the *RootForce* (Gerth et al, 2021) algorithm as target labels *L*_0_ (**Figure 4B**). Each iteration applies the sub-label categorization (**Figure 4D**) from Section 3.5 based on the current approximated target labels *L_i_* (**Figure 4C**) and the gray value CT data *D* (**Figure 4A**).

As shown in **Figure 4F**, during the inference cycle the training and validation CT volumes were assessed with the custom inference algorithm, see Section 6. According to the work by Khoreva et al. [^26^] as well as our experiments, the resulting root segmentation *O_i_* shows improvements over the noisy label data *L_i_*. In the next training iteration, this improved segmentation replaces the previously used target label volume *L_i_*_+1_ = *O_i_*, hence *T_i_*_+1_ = (*D*, *L_i_*_+1_) = (*D*, *N_i_* (*D*)). With each iteration, all sub-steps are repeated, and a new network *N_i+1_* is trained from scratch. A total of three cycles (i \in {0, 1, 2}) of recursive training were iterated. The resulting network *N*_2_ after the last cycle is fixed as final proposal. In Section 7 and Section 8 the results of this last network model *N*_2_ applied on a completely disjoint test set are presented and discussed.

During the training process, the *cosine annealing learning rate schedule* was applied, as proposed by Loshchilov and Hutter (2016). The combination of the learning rate schedule together with *Snapshot Ensembling* (Huang et al., 2017) was investigated, and showed that a schedule with only one cosine annealing cycle and no ensembling was most promising with respect to the application and the data. Thus, our schedule function had the form


(1) $$\eta_{t}=\frac{1}{2}\eta_{0}(1+\cos(\frac{t}{T}\pi )),$$

where η*_t_* ∈ ℝ^+^ is the learning rate calculated at training step *t* ∈ ℕ, η_0_ ∈ ℝ^+^ is the initial learning rate, and *T* ∈ ℕ is the total count of training steps. Moreover, *T* corresponds to the total count of training samples *|D|*, divided by the batch size *b* ∈ ℕ and the update frequency of the schedule *f* ∈ ℕ, *T* = *|D|/bf*. For all iterations the batch size *b* was fixed to *b* = 256 and the update frequency to *f* = 4,000, where *f* is given in batches, i.e., the step index *t* increments every 4,000 batches. At the end of the schedule the model has seen all training samples once, though, the same voxels are seen multiple times, once for each available FoV size.

## 6 Dynamic inference

Since the proposed network architecture (see **Section 4**) only evaluates one voxel (based on its FoV) at a time, the process to segment one complete root volume by iterating through every voxel independently is very time consuming. Additionally, independent voxel evaluations are prone to misclassifications randomly distributed across the volume. Even though such misclassifications can be addressed by adequate post-processing steps, as e.g., by a *connected component analysis* (CCA, see **Section 8.2**), post-processing is usually undesirable and suffers from inflexible thresholds.

Furthermore, the network’s architecture with the above introduced SPP-layer was selected with the dedicated goal of inferring root samples of arbitrary input sizes and scales. Leveraging this advantage with a naïve voxel-wise sequential inference becomes challenging, as it would require evaluating the complete volume multiple times on different scales. This approach would not only increase the inference time dramatically (up to an order of multiple days) but would also require an adequate process to merge the segmentations from different scales into one joint result, which is a non-trivial challenge.

For these reasons a custom inference approach – based on the well-known flood-filling paradigm from classical image processing – was developed to not only speed up the inference of one root volume, but also simultaneously improve the segmentation quality. Hence, in an iterative process, only those voxels are chosen for evaluation, which are most likely to contain a root. This selection of possible root voxels is guided by previously evaluated voxels within a certain neighborhood, hence, the independence of the aforementioned voxel-wise inference can be disrupted. Also, this approach eliminates the need for post-processing as it automatically generates a connected root structure. Furthermore, the results are improved by adaptively changing the size of the FoV which is used for the classification of each voxel into the classes *‘root’* or *‘non-root’*.

**Figure 5:**
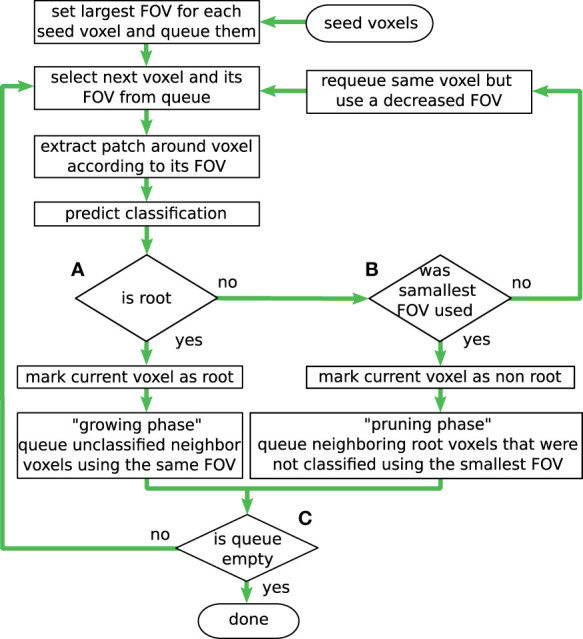
Workflow of the inference algorithm. By iteratively evaluating only neighbor voxels of known roots (‘growing phase’), the algorithm works in a flood-filling manner, by choosing 'yes' at **(A)**. Different sizes for the field of view (FoV) support the trade-off between classification confidence and spatial accuracy. The border of the root is detected adaptively in the ‘pruning phase’ with the most accurate/smallest FoV, by choosing 'yes' at **(B)**. The inference terminates when all queued voxels have been processed **(C)**.

The workflow of the proposed inference algorithm is shown in **Figure 5** and will be discussed step by step in the next sections.

### 6.1 Flood-filling approach

The iterative process starts at the top of the flow graph in **Figure 5** by (manually) choosing a set of initial seed voxels, of which the user is certain that at least one of them belongs to the class *‘root’*. These seed voxels are then pushed into a queue, together with the information that at processing time the largest available FoV size should be used for them. Even though the diagram in **Figure 5** depicts the workflow for a single voxel at a time, multiple voxels can be pulled from the queue at once, to make use of the more efficient batch evaluation. When a voxel is pulled off the queue, its FoV (with radius *r*) from the neighboring voxels is extracted from the volume and prepared as a sample for the network’s input layer. If the network classifies the actual center voxel as *‘root’*, the downwards arrow from diamond (a) (in **Figure 5**) is followed.

**Figure 6:**
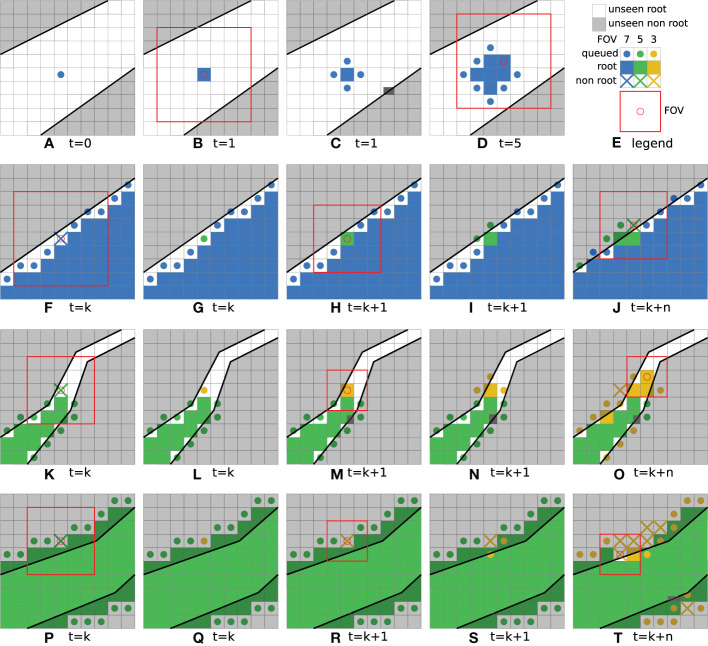
Simplified illustrations of iteration steps during the initial *growing phase* of the seed voxel **(A–D)**, premature edge detection **(F–J)**, tapering root **(K–O)** and *pruning phase* **(P-T)**. **(E)** shows a legend of the used color coding. Note that unseen '\emph{root}' and '\emph{non-root}' areas are depicted as a simplification of the real, non-discretized structures.

In the example depicted in **Figure 6A**, a single seed voxel was chosen. Its queued state is represented by a blue colored disk in the respective voxel. The largest used FoV size in the example has a radius of *r* = 3 voxels and an edge length of *l* = 7 voxels, the related voxels are denoted in blue. **Figure 6E** summarizes the color codings of all depictions. Next, in **Figure 6B**, the network has classified the seed voxel as *‘root’* with the given FoV (drawn as a red box with a red circle in the center voxel) and the voxel is thus represented (labeled) with a filled-in colored blue voxel. The process will then queue all yet unseen neighboring voxels (of the current voxel) for further evaluation as it is shown in **Figure 6C**. This step is denoted as ‘growing phase’, since it fills the queue and allows the detected root structure to grow. While the queue is not empty, and the newly queued voxels are classified to belong to the ‘*root*’ class, these steps are repeated. This is depicted in **Figure 5** by looping back to the top in diamond (c), hence iteratively evaluating the collected and yet unseen neighbor voxels in the queue.

**Figure 6D** illustrates a slightly progressed state of the root volume with a growing amount of already classified as well as queued voxels. In contrast to a naïve brute-force approach, where *each* voxel in the complete CT-volume is evaluated and classified from top to bottom, this process operates similar to a classical ‘breadth-first’ flood-filling algorithm, as it starts at a seed voxel and locally expands in every direction equally, until a stopping criterion is met. Furthermore, to avoid evaluating every voxel in the volume, the goal is to stop this growing or ‘flooding’ process at the boundary of the already detected root structures. However, as these boundaries are not known beforehand, they must be detected as precisely as possible during the expansion process.

### 6.2 Boundary detection

To robustly detect the boundaries between the root structures and the surrounding soil, advantages are made of the possibility to process FoVs of different spatial sizes. It can be assumed that large FoVs will lead to predictions with a high degree of certainty, where the network is confident in its classification, as it has much contextual information on which to base its estimate. Furthermore, it is assumed that small FoVs will lead to predictions which are locally more accurate, since smaller FoVs might detect the exact voxel position of a root-boundary with higher spatial accuracy than a large FoV, as there exist fewer off-center voxels competing for attention of the network. In contrast, a large FoV can contain many root voxels, none of which are in the center position. This effect could erroneously skew the prediction towards the *‘root’* class, especially when those root voxels are located directly next to a center voxel.

Under these considerations, the best achievable boundary detection of the root structures will likely be computed with small FoVs. For this reason, the proposed evaluation process starts with the largest FoV around the seed voxels, since it allows going from the most confident classification (large FoVs) to the spatially most accurate one (small FoVs). Hence, if a voxel is pulled from the queue and is classified as ‘*non-root*’, the size of the FoV is reduced to a smaller radius and the same voxel is pushed in the queue again for further evaluation. This extends the formerly mentioned loop in **Figure 5** by an alternative route, following the ‘no’-arrows from diamond (a) and again from diamond (b), if the currently used FoV is not the smallest one possible.

As depicted in **Figure 6F**, this effect can happen close to the boundary of the root. Here, the currently processed voxel was queued with the largest FoV with radius *r* = 3, and edge size 7. However, in this example the network classifies the voxel as ‘non root’, indicated by a colored cross in the figure. With this classification result, the voxel is inserted in the queue again, now with a smaller FoV. Hence, in **Figure 6G** the next smaller FoV with radius *r* = 2 and edge size 5 is depicted with a green color-coding. The reduced FoV size now helps the network to focus more on the actual center voxel of the FoV. When the voxel is then classified as *‘root’*, the following neighbor voxels will continue to use the smaller FoV, as can be seen in **Figures 6H, I** respectively. If the voxel is still classified as ‘*non-root*’, the FoV size is further reduced as shown in **Figure 6J**. Here a neighbor of the previously discussed voxel was classified as ‘*non-root*’ with a FoV of edge size 5. In this way, the voxels of the boundary layers of the root structures are grown by smaller FoVs. This especially comes into effect for regions in which the gradient of the gray-level values promotes a premature edge detection for larger FoVs.

An equivalent process takes place in regions in which the root structure tapers off too much for large FoVs to correctly classify them. This situation is shown in **Figures 6K-O**. Starting with a FoV of edge size 5, a voxel classified as ‘*non-root*’ is queued again with a smaller FoV of edge size 3, illustrated with an orange color-coding. Using the smaller FoV, the voxel is then correctly classified as *‘root’* and the growing phase now continues along the thinner root structure. The further progressed state in **Figure 6O** shows that this effect can happen with multiple voxels at the same time. Note that the processing order of the queued voxels in these sketches do not follow the standard FIFO order of a queue, but rather the order was chosen to illustrate the idea. During the inference process, the queued voxels are evaluated simultaneously as part of the same batch.

As described above, the ‘growing phase’ leaves the FoV size unchanged between the current voxel and the new neighbors which are pushed in the process queue. The FoVs edge size will only be decreased in areas, where a voxel has been classified as ‘non root’. This approach will carry the high confidence levels of the large FoVs through the already grown root structures and sacrifice it for a higher spatial accuracy only where needed. In this way, the radius of the FoV will decrease more and more until a given lower boundary value of *r* = 1 and edge size of 3 is reached (depicted with an orange color-coding). A classification as ‘non root’ with this FoV, as indicated in **Figure 6O**, will lead to the next phase of the process.Following the downward ‘yes’ arrow from diamond (b) in **Figure 5**, the algorithm now knows with the highest confidence and spatial accuracy, that the current voxel is *not* from the *‘root’* class and marks it as such. However, this level of spatial accuracy cannot yet be assured for the previously classified root voxels. To tackle this problem, a so-called ‘pruning phase’ is initiated in the affected local region. At this point, it is possible that the already grown structure extends over the true root’s boundaries. In contrast to the premature edge detection in the growing phase, the gradients of the gray level values in the CT data can also promote a delayed edge detection for larger FoVs.

**Figure 6P** shows an example with a FoV of edge length *l =* 5 voxels. In **Figure 6Q** the process queues the same voxel again with a smaller FoV with radius *r* = 1 and an edge length *l =* 3, which is the smallest FoV possible. As the voxel is already located considerably across the root boundary, the smaller FoV classifies the voxel as ‘non root’ in **Figure 6R**. Since the smallest allowed FoV was already used, this initiates the pruning phase in that region of the volume, which allows to double check the currently proposed segmentation by the larger FoVs.

Under these circumstances it may happen that neighboring voxels, which have previously been classified as *‘root’* within a larger FoV, will be re-evaluated using the smallest FoV in later iterations. If the re-evaluation reveals misclassifications by the previously used larger FoV, the pruning process is successful, and the root boundary is corrected and shifted back by updating the classification result at that voxel from ‘*root*’ to ‘*non-root*’.

**Figure 6S** shows this effect of requeuing a voxel sample with a FoV of edge length *l =* 3, where previously a FoV of edge length *l =* 5 has predicted the class *‘root’* in an earlier iteration. When the re-evaluation detects the opposing class, the process is repeated in such a way that it again follows the downward arrow in **Figure 5** from diamond (b), resulting in another alternative loop.

Even though it only affects voxels, which were classified as *‘root’* by a FoV larger than the smallest one, this path also results in a flood filling operation. Thereby, the boundary of the root is shifted slightly backwards to where the most spatially accurate FoV detects it to be. This is shown in **Figure 6T**, where in a slightly progressed state of the process, more voxels have been pruned and queued for re-evaluation. If the re-evaluation step confirms the previous result, the pruning phase terminates, and no new neighbors will be pushed to the queue. One example of this possibility is the lower neighbor of the originally discussed voxel in **Figure 6P**. If all voxels in that region reach this state, the segmentation of the local root structure can be considered complete.

### 6.3 Parallelization

Through the usage of the queue data structure, a parallel implementation of the inference evaluation processes as well as processes concerned with the enqueing of new neighbors is possible. Thus, multiple growing and pruning phases can be performed at the same time in different regions in the CT volume. This is also indicated in the lower right corner of **Figure 6T**, where a new pruning phase has started on the other side of the root. When the queue is empty, the inference algorithm will terminate for the whole volume.

## 7 Experiments and results

Using the data (Section 3) and the methods described above, a CNN was trained, using a SPP-layer (Section 4) to improve the scale invariance during the segmentation of root structures in CT data. A weakly supervised training (Section 5) loop was applied to improve noisy label data through the generalizing capabilities of neural networks. Using three training loops, three networks were trained from scratch, whose progress is presented in Section 7.1.

To create an adequate data set, a sub-labeling technique (Section 3.5) was applied, which categorizes each label into a more specific sub-label, by differentiating between content types and location in each training sample. This approach enables a fine-grained control over the diversity and difficulty of the training samples. Even though the impact of this sub-labeling is difficult to quantify, in Section 7.2 and Section 8.1, results are presented and discussed with respect to classifying the plant pot (included in the CT scans, see **Figure 1**) which was frequently misclassified by networks trained *without* the sub-labeling technique.

For the testing phase, a novel inference approach was developed (Section 6), which operates in a flood-filling (or volume growing) scheme and can dynamically adapt the size of the field-of-view (FoV) of the input samples to achieve an overall high-quality segmentation. The results achieved with the proposed dynamic inference are compared with a naïve (brute-force) inference algorithm as well as the reference *RootForce* segmentations (Section 3).

### 7.1 Network training iterations

**Figure 7:**
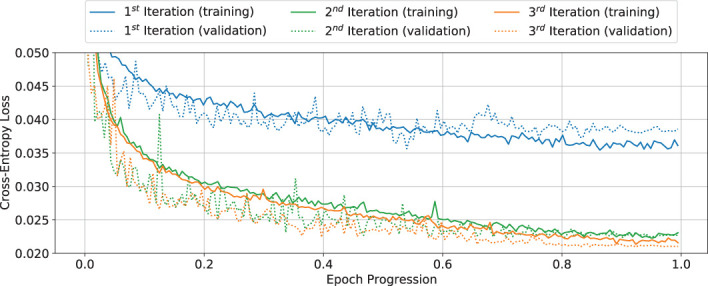
Showcase of the improvement, measured by the cross-entropy loss function, for the training (solid) and validation (dotted) curves over three weakly supervised training iterations. Curves show original data points; no smoothing was applied.

**Figure 7** depicts the loss curves for the training (solid) and validation (dotted) data set over all three training iterations. A vast improvement from the first to the second iteration can be observed, which also corresponds to an improvement of the label data available for successive iterations. The improvement from the second to the third iteration is significantly reduced. Hence it can be concluded that the chosen net architecture (Section 4) has exhausted its ability to generalize based on the available small training data set (Section 3.2), as the training has reached a minimum. Hence, the training was stopped after the third iteration and the network was stored as ‘final network’ *N_2_*.

### 7.2 Test setup

**Figure 8:**
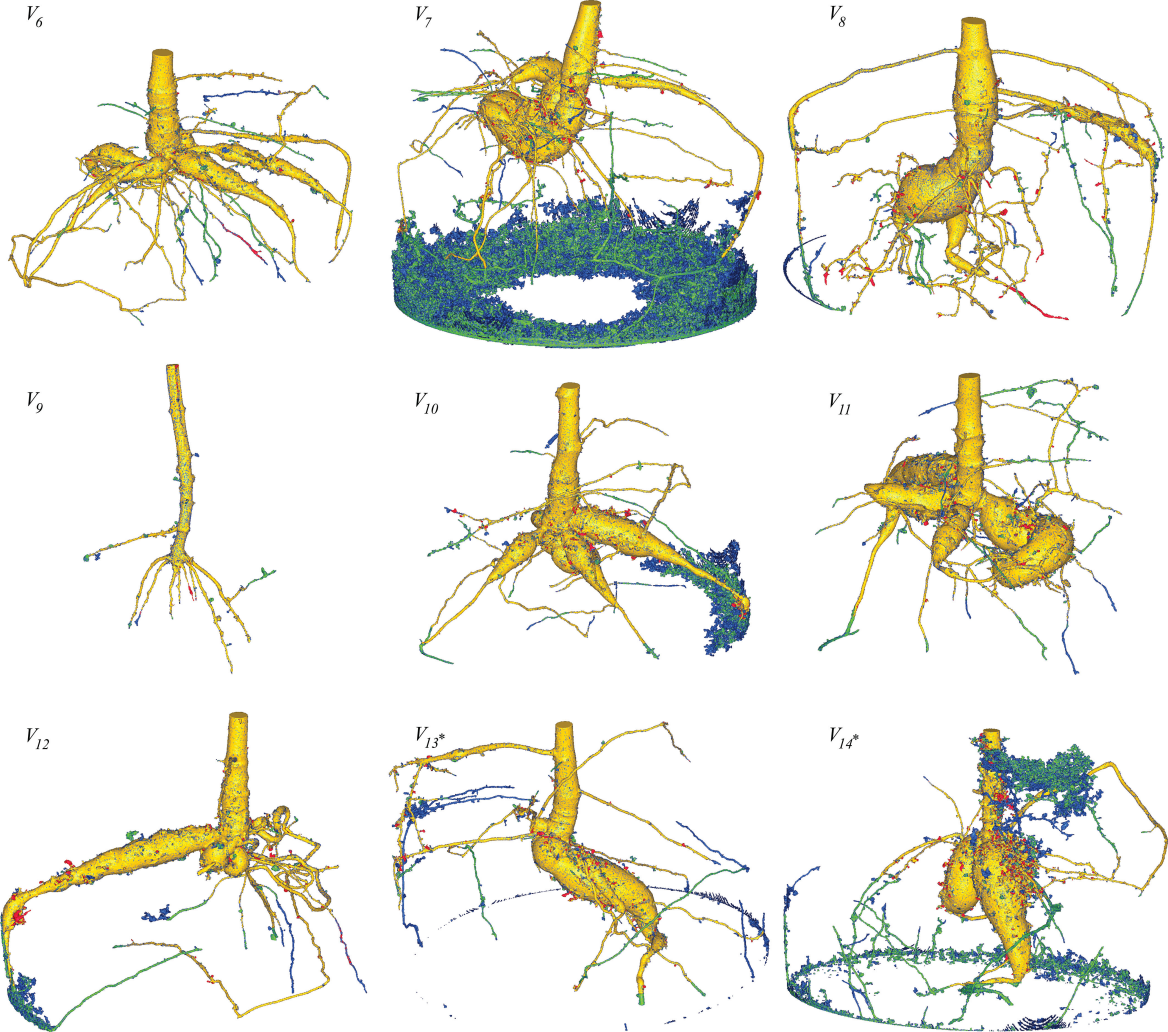
Fused renderings of the segmented 3D root systems from the test data, obtained from *RootForce* and the proposed DCNN *N_2_*. The two volumes marked with an asterisk ∗ had an additional post-processing step (see text). Colors decode the segmentation methods. Yellow: intersection between voxels segmented by the proposed DCNN with a 50% threshold and *RootForce*. Green: additionally detected root branches by the DCNN with a 50% threshold. Blue: further additionally detected root branches by the DCNN with a 20% threshold. Red: root segments only discovered by *RootForce*.

Each of the three evaluated inference methods (*RootForce*, naïve inference, dynamic inference) apply an effective volume mask to reduce the region that needs processing; These are described here for reference:

(1) Firstly, *RootForce* automatically detects the *‘air-soil’* transition at the top of the CT-volume and excludes all *‘air’* voxels above from further assessment. Some of the CT’s bottom layers are excluded as well, since the possible aggregation of water at the bottom of the pot causes higher attenuation coefficients and after the CT reconstruction, water aggregation yields CT gray level values similar to those of root structures. Hence, *RootForce* tends to misclassify humid soil as *‘root’* if these layers would not be excluded. *RootForce* is able to estimates the maximal layer depth dynamically, which corresponds to the humid soil humidity.
(2) For the *na*ï*ve inference* algorithm with the DCNN, the plant pot mask is applied, which was introduced in Section 3.5. Thereby, the classification of many background voxels was omitted, which saves approximately a third of the necessary computation time.
(3) For the proposed *dynamic inference*, only the masked bottom of the pot was excluded. As mentioned in **Section 3.5**, one goal was to develop and train a network which does not misclassify the plant pot voxels as *‘root’* voxels. To test this, the test data volumes were inferred without any mask of the pots, and it was found that the mantle area of the pot is successfully absent from the segmentation. However, the bottom of the pot was falsely included in the segmentation every time a root branch reached down far enough. Hence, for the presented results the pot bottom was masked during inference to have a fair comparison with the other methods, as extra voxels from the pot bottom would overshadow the voxel count of the small roots and render a quantitative comparison more challenging. Furthermore, when visualizing the 3D structure (see **Figure 8**), the pot bottom practically eliminates the contrast needed to recognize small roots in the foreground.

Two exceptions were made for the dynamic inference results, which are marked by an asterisk in **Figure 8** and **Table 2**, namely volumes *V*_13_ and *V*_14_. Here the volumes were initially inferred with a mask that left the upper ridge of the pot bottom accessible to the flood-filling operation. This led to a segmented ring in *V*_14_, where the upper ridge around the pot was falsely classified as *‘root’*. This in turn allows the network to find additional small roots that otherwise would not have been connected to the main root-structure, as the mask of the pot acts as a natural barrier to the detection of root branches that first grow to the bottom of the pot and then *reach back up*. In these exceptions, the additionally detected small roots were preserved, while the ring artifacts belonging to the pot bottom were deleted in a post-processing step with another mask. By this approach, the ability of the proposed network to classify roots that were *not* detected by the reference method *RootForce* is better demonstrated. Moreover, these upward growing roots are a prominent example of the network correctly classifying the pot mantle as *‘non-root’*, since long segments of those branches practically stick directly to the plant pot. While this results in a disconnected segmentation structure, it enables a more sensible comparison of the voxel counts.

**Table 2:** Comparison of root voxel counts for the test data from the reference method *RootForce* (S_RF_) and the proposed DCNN approach with thresholds of 50% and 20% on the class probabilities (S_N50_, S_N20_).

Color	Domain	Voxel counts								
		V_6_	V_7_	V_8_	V_9_	V_10_	V_11_	V_12_	V_13*_	V_14*_
Red	S_RF_ \ S_N50_	90,904	106,012	111,695	6,303	86,306	77,135	74,401	82,957	235,468
Yellow	S_RF_ ∩ S_N50_	3,091,392	5,124,173	6,743,898	229,747	4,460,024	4,943,356	1,845,211	3,563,454	4,973,605
Green	S_N50_ \ S_RF_	79,988	4,944,945	130,176	17,067	198,783	88,672	88,683	69,804	500,967
Blue	S_N20_ \ (S_RF _ ∪ S_N20_)	51,126	2,213,364	76,944	16,357	163,549	53,569	56,548	92,750	337,683

The two volumes marked with an asterisk ∗ had an additional post-processing step (see text).

In contrast to the reference segmentation method *RootForce*, the proposed DCNN approach is able to detect and segment plant structures *above* ground. Nevertheless, the comparison has been restricted to root regions below ground.

For the initialization of the proposed dynamic inference algorithm, single seed voxels were used, which were a-priori known to be part of the *‘root’* class. These positions are usually not known exactly, however, they can be found *via* thresholding within the main plant stem. Alternatively, a small set of voxels enclosing a region in the volume that is likely to contain a root can be used as seed voxels.

### 7.3 Visualization

**Figure 9:**
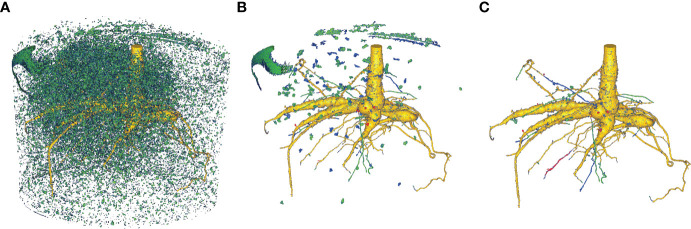
Comparison of the DCNN *N_2_* segmentation results from a naïve inference approach and the proposed dynamic inference algorithm for volume *V*_6_. **(A)** Naïve inference with FoV *r* = 5, **(B)** naïve inference with FoV *r* = 5 + CCA, **(C)** proposed novel dynamic inference. Color-coding is identical to **Figure 8**. Yellow: intersection between voxels segmented by the proposed DCNN with a 50% threshold and *RootForce*. Green: additionally detected root branches by the DCNN with a 50% threshold. Blue: further additionally detected root branches by the DCNN with a 20% threshold. Red: root segments only discovered by *RootForce*.

In the following, the test data segmentations are presented with a visual comparison, based on a fused rendering of the 3D structure of the proposed dynamic inference method using *N_2_* as DCNN approach and the reference method *RootForce* (see **Figure 8**). Additionally, the segmentation results of the dynamic inference are visually compared to the segmentation of the same network using a *naïve inference* algorithm, as depicted in **Figure 9**.

The affiliation between classified voxels and the underlying segmentation approach is denoted *via* set notation. Firstly, the set of all voxels segmented by the *RootForce* approach is denoted as *S*_RF_. Secondly, the set of all voxels segmented by the proposed network *N*_2_ (from the third training iteration) and using a threshold *θ* = 50% on the class probabilities (the network has a confidence above 50% that a voxel belongs to the *‘root’* class) is referred to as *S*_N50_. Finally, the network segmentation using a threshold of *θ* = 20% is denoted by *S*_N20_.

The voxel coloring scheme in **Figures 8**, **9** decodes the segmentation method and is denoted in set notation in **Table 2**, where the ‘\’ sign denotes the difference of two sets (e.g. ‘A\B’ translates to ‘set A without set B’): *‘yellow’*, *S*_N50_ ∩ *S*_RF_ indicates the intersection between all voxels segmented by the proposed DCNN *N_2_* and the voxels proposed by *RootForce*; *‘green’*, *S*_N50_ \ *S*_RF_ denotes *additionally* detected root branches by the DCNN; *‘blue’, S*_N20_ \ (*S*_N20_ ∪ *S*_RF_) relates to further additionally detected thin root branches with the lower 20% threshold; *‘red’, S*_RF_ \ *S*_N50_ indicates root segments, which are *only* discovered by *RootForce*. To obtain a quantitative comparison of the investigated methods, in **Table 2** the voxel counts for each color domain are listed separately.

For visualization purposes, the opacity of a one voxel thick layer around the yellow surface was set to zero in both **Figures 8**, **9**. However, no root branches were deleted, and hence the qualitative extent and structure of the root system is not altered. This eliminates hollow red or green structures which wrap around the yellow domain and thereby reveals the true extent of the segmentations intersection. Nevertheless, this effect yields some apparent discrepancies in the voxel counts in **Table 2** when comparing it to the figures, as the voxel counts still include these hidden voxels. This effect is later discussed in Section 8.1. A similar procedure could have been applied to the surface of the blue regions. However, since the overlapping voxels are less significant in their count, and thus do not disturb the visualized information, this was neglected.

## 8 Discussion

In Section 8.1 the segmentation results on the test data set are compared between the proposed DCNN *N_2_* segmentations and the analytical reference approach (*RootForce*). Differences between the naïve and dynamic inference approaches are discussed and highlighted in Section 8.2. Performance considerations for the inference methods are outlines in Section 8.3. Finally, the shortcomings of the proposed methods and an outlook for possible improvements are presented in Section 8.4.

### 8.1 Comparing to the reference

**Figure 8** shows the segmented root systems of all test volumes (*V*_6_ – *V*_14_). Generally, in comparison the segmentation results of the proposed DCNN *N_2_* approach and the analytical reference method (*RootForce)* are quite similar, and most of the root structures are detected by both algorithms (*yellow* voxels in **Figure 8**). However, in all test volume the proposed deep neural network detected numerous additional thin root branches with the 50% threshold (*green* voxels in **Figure 8**), and even more with the 20% threshold (*blue* voxels in **Figure 8**), which have not been discovered with the reference approach *RootForce* (of which exclusively detected voxels are *red* in **Figure 8**).

As one major finding, the *quality* of the root surface is increased using the proposed deep neural network approach. This effect is noticeable when focusing on the storage roots and comparing the count of different colored ‘blobs’, which are mostly misclassifications, attached to the root surface. For most of the test volumes, the red colored blobs outnumber the green and blue blobs. Thus, even with the low confidence threshold (*θ* = 20%), the proposed DCNN approach yields competitive results.

The achieved voxel counts of detected root structures are listed in **Table 2** and can be used for a quantitative assessment of the different colored domains. The higher segmentation quality of the proposed approach is hinted at by an increased count of detected root voxels. However, there is a noticeable lack of visible, red-colored voxels in relation to visible green- and blue-colored voxels in **Figure 8** and the comparatively small difference in voxel counts in **Table 2**. This results from *excluding* voxels for an unbiased visual comparison, as explained in Section 7.3.

The excluded voxels of the red domain (voxels exclusively detected by *RootForce*) are concentrated along the surface of thin root branches, while *excluded* voxels of the green and blue domains (voxels exclusively detected by the DCNN) are scattered on the surface of large storage roots in similar quantity as the individual voxels which are still visible as dark dots on the yellow surface. Most voxels exclusively found by *RootForce* (*red*) widen the commonly detected (*yellow*) root branches, while voxels exclusively found by the proposed network approach (*green, blue*) extend the structure by new thin root branches. In terms of a qualitative comparison, there is a difference between detecting more surface voxels (like *RootForce*) versus detecting more root branches (like the DCNN). Contrary to the detection of wider branches, previously unknown root branches yield additional and desired information about the overall structure of the investigated plant root systems, especially when considering the ambiguity of ‘correctly’ discretizing a continuous surface with voxels.

The most noticeable flaws of the proposed DCNN approach are the large, misclassified regions in the test volumes *V*_7_, *V*_10_, and *V*_14_. These regions prohibit a useful comparison *via* voxel counts, since the misclassified voxels outnumber the ones that make up the small root branches, and hence cannot be easily separated from the true root system. Investigation of the cause for such errors showed an altered gray level profile in the affected areas, which was caused by higher water content in the soil and lead to an increase of the gray level values in the CT reconstruction. Unfortunately, these increased gray levels are very similar to the gray levels of many root structures. This observation indicates that the trained network has been fine-tuned to a specific soil mixture with a certain water saturation level which results in a characteristic gray level distribution adjacent to the plant. Hence, the obvious cause of this observed effect is the identical scanning parameters for the CT data acquisition of training data.

Focusing on the segmentation results of the DCNN with the lower threshold (*θ* = 20%, ‘blue’), further significant improvements in detecting thin root branches can be observed. Using the lower threshold, the network detects the same root voxels as when using the higher threshold (*θ* = 50%), and further extends the segmentation. Many of the test volumes (see **Figure 8**, **Table 2**) have a similar amount of green- and blue-colored root branches, meaning using the lower threshold (*θ* = 20%) resulted in nearly twice the amount of detected root voxels. A few small root branches are also detected by the reference method as well as the proposed network with *θ* = 20%, but not with the 50% threshold. Two examples of this are in volume *V*_6_ (mainly red and partially blue branch, growing from the center to the lower right) and in volume *V*_12_ (furthest branch to the right, growing downwards).

Generally, the lower threshold (*θ* = 20%) also comes with the cost of an increased count of misclassified voxels. However, the effect is more subtle than expected, since most misclassifications happen in regions, where the network already struggles with a higher threshold (*θ* = 50%), e.g., at the humid sediment in volumes *V*_7_, *V*_10_, and *V*_14_. Though, misclassifications of soil lumps sticking to the root surface are more frequent with lower threshold compared to *RootForce.*

Some roots that follow the outer edge of the plant pot, as depicted for volumes *V*_8_, *V*_13_ and *V*_14_, highlight the success of the proposed and employed sub-labeling technique. In such situations, other networks models with the same network architecture and trained *without* the sub-labels, have frequently initiated the inference algorithm to follow the plant pot (by ‘flood-filling’) as well. This argument can also be made for the bottom of the plant pot as a counter example, since it was not focused on in training by the sub-labels (see Section 3.5). Note that the pot bottom was excluded from the segmentations in **Figure 8** by a post-processing step as explained in the previous section.

It can be argued that the problem of the misclassification of the base of the pot shows the advantage of properly applying the proposed sub-labeling technique, as it allowed for exact control over samples from the pot mantle but not the pot bottom (see Section 3.5). More precisely, specifically choosing samples from the approximated segmentations of the pot mantle to augment the training data, resulted in successfully teaching the network to *not* recognize the *‘pot’* voxels as *‘root’* voxels. However, due to the complicated geometry of the pot bottom, no reference segmentations were available from which specific samples could augment the training data. Therefore, the alternative of randomly sampling from the bottom volume to statistically include challenging samples in the training data was used. Unfortunately, this did not focus learning on such samples sufficiently and hence was not successful in teaching the network to identify voxels from the pot bottom as ‘*non-root*’. This problem could be alleviated by possibly increasing the training samples from the pot bottom. However, this also could alter the proportion of multiple sub-labels to an unknown degree and therefore opposes the principle of fine-tuning the composition of the training data.

### 8.2 Comparing to the naïve inference

As described in **Section 6**, the *naïve algorithm* iterates through every voxel in the volume independently. This approach has the disadvantage that mostly ‘*non-root*’ voxels are queried, since they constitute most voxels in a volume and thus, waste expensive computation time. Moreover, this approach is more vulnerable to misclassifications as the deep neural network never connects the queried results within one volume, leading to a cluttered 3D structure (see **Figure 9A**). Here, a helpful post-processing step is the application of a ‘connected component analysis’ (CCA), which extracts the largest detected structures and removes most of the clutter. In **Figure 9** the segmentations from the proposed DCNN *N_2_* from the third training iteration are depicted, comparing the naïve and dynamic inference methods. To aid the visualization, the reference segmentation (*RootForce*) is included in the illustrations for an identical color-coding to **Figure 8**. In **Figure 9A**, the raw output of the naïve inference algorithm is depicted for Volume *V*_6_. It is obvious that there exists a lot of clutter, namely misclassifications, generated by the network (depicted as *green*- and *blue*-colored voxels), which obscure the view onto the root structure in the center.

**Figure 9B** shows the result after applying the connected component analysis (CCA) which deletes all structures consisting of less than 1,000 connected voxels. This results in only some larger clumps of soil in the segmentation. Some large, misclassified structures are part of the pot (top left) and some topsoil sticking to the pot (top right, background). Also, small root structures that have been detected by the network, become visible in this view. Those are found around the root in the lower half of the illustration. The segmentation result of the proposed dynamic inference is depicted in **Figure 9C**. Since the proposed dynamic inference approach automatically generates only one single connected component, unwanted structures in the volume are automatically discarded. However, this comes at the cost of possibly undetected root sections, which *could* have been correctly classified by the network using the naïve inference (cf. **Figure 9B**) but remain hidden for the flood-filling approach. For example, in **Figure 9B** there are three thin green root branches visible in the foreground (horizontally centered). In **Figure 9C** one of them is missing in the segmentation using a threshold of \theta = 50% but detected by the lower 20% threshold (colored in blue). Another branch (colored in red with some blue depicted in **Figure 9C**), indicates that only the reference method (*RootForce*) and the deep neural network with threshold *θ* = 20% was able to detect it, while the same branch is colored *yellow* in **Figure 9B**, indicating that the network with threshold *θ* = 50% can detect it as well when it is not blocked off by the flood-filling process.

In conclusion, an obvious tuning parameter is *θ*, thresholding the class probability of the network output. Lower thresholding (e.g., *θ* = 20%) is able to segment thinner and finer roots, however, is also susceptible to more misclassifications. For the naïve algorithm, this means also more unwanted large, connected components which are harder to separate from the desired root branches, as can be seen in **Figure 9B** by the additional blue soil clumps. The segmentation of the dynamic algorithm is affected more positively by a low threshold, since it increases the connectivity of the root components as described above, while other parts of the segmentation are barely altered. This, on one hand, leads to more detected thin roots, which are connected to the main-root structure, but on the other hand, leads to a slightly rougher root surface on a few branches. This can be seen in **Figure 9C** by the thin blue root branches and the blue dots along the *yellow* surface respectively.

We also note that the naïve inference only uses one FoV for all queries, while the proposed *dynamic inference* adaptively changes the radius *r* of the FoV. A FoV with edge length *l =* 5 was applied for the naïve inference. Using larger FoVs with a constant radius would lead to less, but still obstructing, clutter in the raw segmentation and would furthermore fail to detect thin root branches. When investigating the effect of *varied* FoV sizes of the dynamic algorithm, it becomes apparent that most voxels are evaluated with the largest, initial FoV size. Adaptation to smaller sizes happens only at the direct root boundaries or very thin root structures with small local voxel counts. Hence, the effect is not statistically significant for the voxel count. However, using only larger FoVs with the flood-filling approach misses most of the thin roots and yields thicker root structures overall, due to the missing pruning step.

Furthermore, for the naïve inference the count of root segments kept after the CCA is dependent on a hyper-parameter. This could be set in such a way that only the largest connected component, or components which fulfill known geometric requirements such as elongation restrictions, are kept in the volume. This could yield segmentations, which closer resemble the segmentation of the proposed dynamic algorithm. However, choosing adequate criteria to discard disconnected components becomes challenging since small and thin roots are easily caught erroneously when trying to declutter the segmented volume.

Some of the soil lumps in **Figure 9B** can also show up within the dynamic inference, if its initialization consists of *guessing* where possible root voxels are located in the volume, e.g., by selecting a small surface of seed voxels in the volumes center. As long as the root structure crosses the surface, all parts of the roots will be detected as shown in **Figure 9C**. If some single soil lumps – depicted in **Figure 9B** – happen to cross this surface, they would be included in the segmentation of the dynamic inference as well.

### 8.3 Performance

In the following, the evaluation times of the different inference methods are compared. Improvements are mainly achieved with respect to the naïve method, as the *RootForce* algorithm is based on classic image processing methods whose run-time is highly optimized.

The *RootForce* algorithm always needs the same time duration to evaluate a volume of a fixed size. For our test volumes (1024 × 1024 × 900 voxels, see Section 3) this amounts to approximately 10 minutes on an Intel Xeon CPU (E5-2620v4@2.10GHz) with 256 GB system RAM.

Evaluation with the naïve method also takes the same time for a fixed volume size. However, the run-time significantly depends on the used FoV size. The limiting factor is the VRAM used on the graphics card for each batch. For sequential processing on one GeForce GTX 1080 Ti GPU, conducted measurements with FoV edge lengths of *l* = 5 and *l* = 15, allowed for batches of size 2^14^ = 16384 samples and 2^9^ = 512 samples respectively, and resulted in evaluation times of roughly 120 and 3,000 minutes respectively.

The run-time of the dynamic inference algorithm is dependent on the amount of connected root voxels, which are present in the volume, whereby the connectivity is decisively dependent on the used threshold on the class probability. A more subtle influence comes from the structure of the root itself. Since more voxels are queued simultaneously during the flood-filling of thicker roots, the evaluation can leverage a better parallelization *via* the batch size. Contrary, in thin roots, voxels are queued more sequentially which results in smaller batches per query. For a maximal speedup, the parallel processing capabilities mentioned in Section 6.3 are utilized. With four GeForce GTX 1080 Ti GPUs (in one computation node), the smallest root system (*V_9_*) can be segmented in 5 minutes, the largest root system (*V_8_*) needs up to 25 minutes. Misclassifications as in *V_7_* also cost extra time, which in this case amounted to 36 minutes.

For comparison we assume the best conditions for the naïve algorithm, i.e., inference with FoV of edge length l = 5 and perfect speedup for parallelization (i.e., the measured processing time of 120 minutes is divided by the count of used GPUs). When using four GPUs, the naïve algorithm can be quicker for exceptionally large root systems, however for our testing volumes this was not the case. When using more than four GPUs (in multiple computation nodes) the naïve algorithm will surpass the dynamic algorithm, since the communication overhead for parallel processing of the naïve algorithm is trivial and hence, has an advantageous scaling property.

However, when scaling with respect to volume size, the dynamic algorithm will have an advantageous scaling property, since the fraction of root voxels to volume size grows slower than the total voxel count in the volume. Therefore, less queries are required in the dynamic algorithm to process a large volume. Even more so, if no mask is available to generously reduce the processed region for the naïve algorithm (see Section 7.2). Scaling with respect to volume size applies to larger objects or objects of the same size being scanned with a higher voxel resolution.

### 8.4 Shortcomings and outlook

One general complication of evaluating the quality of the proposed set of methods is the unobtainable, pristine ground truth data of the root structure. Even manual annotation would most likely not yield the desired quality, due to the error-prone fine structures and low contrast gray levels (see Section 3.1). Hence, the exact structure and total voxel count of the true root system is never known, and thus, it cannot be stated how many root branches are left undetected or are falsely segmented. Furthermore, it is challenging – if not even impossible – to decide whether undetected small roots are the result of a lack in generalization capabilities of the proposed deep network model or are due to too many false negative samples in the training data set or changes in the scanning system. Essentially, this effect blurs the line when deciding at which point the deep neural network starts overfitting to the true data distribution, as the network model possibly learns unknown characteristics of the flawed training data set, which can then not be called out by the validation and test data sets inheriting the same flaws. In this case, ‘successfully’ learning generalizations of adequate features on the flawed data might be equivalent to a data set specific adaptation, i.e., overfitting, when considering the unknown ground truth data. Nevertheless, typical cues for overfitting do not show up on the available training curves for the data sets (see Section 7.1).

An obvious shortcoming of the investigated approach is the problem of the bottom of the plant pot. Hence, for future extensions and applications, it is advisable to use plant pots with either a more trivial geometry or obtaining an alternative segmentation of the plant pot for the sub-labels (see Section 3.5) and thus focus more on the negative samples during the training. Alternatively, if the range of future application is limited, using a segmentation of the plant pot as mask to prohibit network queries outside the sediment region is also a viable option, since using such a mask during the inference run has no disadvantages for the computation time (see Section 8.3).

The comparison between the proposed dynamic inference and the naïve inference algorithm shows that inference by flood-filling can be a drawback if the root structure is not one single connected component as seen from a certain threshold. Basically, root branches which would be detected by the deep network model will not show up in the dynamic inference segmentation, when the branch is by accident disconnected from the main root structure. Increasing the connectivity of the detected root structures is equivalent to an increase in the network’s fidelity, which requires a training data set of high quality. If increased computation times can be handled or tolerated, an alternative approach could be to use the naïve inference with a conservative CCA as post-processing (see **Section 8.2**). The remaining components in the volume could be separated into soil lumps and root branches by a shallow learning model and basic features computed from the geometry of the connected region.

The most significant disadvantage of the proposed DCNN-based segmentation approach is the lack of diverse CT scans of roots. All described experiments were carried out on scans of the same (*Cassava*) plant type, grown in the same soil mixture and captured with the same scanning parameters and CT scanner (see **Section 3**). Alternating any of these influencing factors will considerably change the problem statement in the form of the gray level distributions. This would limit the current network model in scope and usefulness, as the discussion on the humidity levels in the sediment already indicated (see **Section 8.1**). Generalizing the proposed network at this level will require a careful composition of diverse CT scans for the training procedure, and most likely a larger network architecture. Other possible approaches could be to train a generative model, to map arbitrary CT scans of root systems to a common generalized latent space, in which a simple model like ours could be used for final segmentations.

A drawback of the dynamic inference algorithm is that it was specifically developed to work with single voxel predictions from the DCNN. This requires significantly more forward passes through the neural network to evaluate every voxel, than modern *image-to-image* DCNNs, like the U-Net (Smith et al., 2020; Soltaninejad et al., 2020), could achieve. Nevertheless, a trade-off between fast computation time (e.g., by using U-Net architectures) and carrying semantic context between individual predictions (e.g., by using flood-filling procedures) has to be made. A promising direction for future research was proposed by Januszewski et al. (2018) (and recently evaluated by Gruber et al. (2021)) who integrated the flood-filling approach directly into an image-to-image DCNNs framework called “Flood-Filling-Networks”.

## 9 Conclusion

In this work, we presented a modified deep convolutional neural network architecture including a spatial pyramid pooling layer paired with a dynamic inference algorithm and a sub-labeling method, which combined are capable of segmenting 3D plant root-structures automatically in CT reconstruction volumes. An analytical segmentation algorithm was used as reference and baseline, whose outcome was also used for a weakly supervised training scheme of the proposed network model. By incorporating the spatial pyramid pooling layer in the network model, the detection and segmentation of arbitrarily sized root samples in different scales was enabled. Furthermore, the use of sub-labels was introduced in the training phase to extract the most essential samples from the available sparse training data. Thereby, the learning process can be guided to find a robust optimum. To infer large CT-volumes of plant roots within reasonable time scales, a dynamic inference algorithm was presented which operates in a flood-filling (or volume growing) manner and adapts the sample size of each query to yield a high-quality segmentation.

The achieved results of the novel root-structure segmentation approach were qualitatively (by visualization) and quantitatively (by voxel count) compared with the analytical reference segmentation algorithm as well as by comparing the proposed dynamic to a naïve inference procedure. It was shown that the proposed novel delineation approach for root structures shows a significant improvement by segmenting previously hard to detect thin and fine root branches. However, some root branches are still left undetected with the applied weakly supervised learning approach, as not all real-world (“in the wild”) possibilities have been considered in the available training data yet. Nevertheless, the proposed approach shows the advantage that inference of new volumetric root data is applicable without pre- or post-processing steps and has an advantageous scaling property of computation time for larger volumes.

In summary we conclude that the proposed novel deep-learning based segmentation approach for root-structures in CT-volumes improves and extends the possibilities of an analytical reference method, by detecting much more fine and small root branches.

## Data availability statement

The data analyzed in this study is subject to the following licenses/restrictions: Some of the CT data sets of the roots can be obtained from the corresponding author SG. Requests to access these datasets should be directed to stefan.gerth@iis.fraunhofer.de.

## Author contributions

JA proposed the dynamic inference and sub-labelling, did the implementations and generated the balanced data set, conducted the training and inference experiments of the DCNN, and – together with RG – drafted, wrote, and finalized the manuscript, including the graphics. RG supervised the implementation of the DCNN, brought in ideas about 3D flood-filling, helped with the evaluation and interpretation of the experiments, and – together with JA – drafted, wrote, and finalized the manuscript, including the graphics. NW suggested the core concept for the deep learning approach, generated the label date using *RootForce*, and supervised the implementation of the DCNN in the first phase. JC organized and performed the CT scans of the plants, and provided knowledge about the *RootForce* approach. NU as an expert in CT-data analysis gave advice to the team about the CT-scanning, and the data interpretation, he supported the organization of the test specimens for the measurements. TW supported and guided the concept and did the final review and editing of the manuscript. SG provided the idea of the experiments combining deep learning with CT-based plant-root analysis, suggested *RootForce* to generate training data and was also deeply involved in its development. All authors contributed to the article and approved the submitted version.
